# Combination therapy with mineralocorticoid receptor antagonists and SGLT2 inhibitors versus SGLT2 inhibitor monotherapy in chronic kidney disease: an updated meta-analysis of randomized controlled trials

**DOI:** 10.1186/s12882-025-04710-2

**Published:** 2025-12-22

**Authors:** Shaikh Muhammad Daniyal, Hareem Ajaz, Minahil Riaz, Naveen Murad Khatoon, Zunaira Aftab, Habiba Tauqir Gondal, Isbah Gul, Mahwish Sarwar, Fizza Batool, Syeda Laiba Fahim, Amna Noor, Ayan Khalid, Danish Ali Ashraf, Romal Jabarkhil

**Affiliations:** 1 Department of Medicine, Dow University of Health Sciences, Karachi, Pakistan; 2 Department of Medicine, TruGift Health LLC, Wilmington, DE USA; 3 Department of Medicine, Ningarhar Regional Hospital, Jalalabad, AF Afghanistan

**Keywords:** Chronic kidney disease, Sodium-glucose co-transporter 2 inhibitors, Mineralocorticoid receptor antagonists, Aldosterone synthase inhibitors, Urinary albumin-to-creatinine ratio

## Abstract

**Background:**

Chronic kidney disease (CKD) affects millions worldwide and poses a major healthcare challenge. While both sodium-glucose co-transporter 2 (SGLT2) inhibitors and mineralocorticoid receptor antagonists (MRAs) or aldosterone synthase inhibitors (ASIs) have shown individual benefits in CKD, data on their combined use remain limited. This study examined the safety and efficacy of combining an SGLT2 inhibitor with an MRA or ASI compared to SGLT2 inhibitor monotherapy.

**Methods:**

A comprehensive search was conducted on PubMed, Cochrane (CENTRAL), ScienceDirect, and Embase for randomized controlled trials (RCTs) comparing combination therapy with an MRA or ASI and an SGLT2 inhibitor with SGLT2 inhibitor monotherapy in patients with CKD. Continuous and dichotomous outcomes were pooled as Mean Difference (MD) or Risk Ratio (RR), respectively, with 95% Confidence Intervals (CIs) using Review Manager (v5.4.1.), and heterogeneity was assessed using the I² statistic.

**Results:**

Five RCTs (*n* = 808 patients) were included. Combination therapy significantly reduced albuminuria (MD: -32.82% [95% CI: -39.16, -26.48] %; *p* < 0.001), systolic blood pressure (MD: -5.02 mm Hg [95% CI: -6.95, -3.08] mm Hg; *p* < 0.001), and estimated glomerular filtration rate (MD: -2.59 mL/min/1.73 m^2^ [95% CI: −3.78, − 1.40] mL/min/1.73 m^2^; *p* < 0.001). While the risk of hyperkalemia was higher (RR: 1.91 [95% CI: 1.10,3.33]; *p* = 0.02) with combination therapy, no significant differences were found in serious adverse events (RR: 0.95 [95% CI: 0.57,1.59]; *p* = 0.86).

**Conclusion:**

The combination therapy provides added renal and cardiovascular benefits in CKD without increasing serious adverse effects. These findings support its superior therapeutic potential and highlight the need for large-scale, prospective studies to assess long-term effects on hard renal and cardiovascular outcomes.

**Prospero registration:**

CRD420251106173.

**Supplementary Information:**

The online version contains supplementary material available at 10.1186/s12882-025-04710-2.

## Background

Chronic kidney disease (CKD) is a growing global health concern, impacting over 800 million individuals worldwide [1]. Characterized by persistent kidney structural and functional impairment, CKD is projected to become the fifth leading cause of death by 2040 [2]. Recent pharmacologic advances have significantly transformed the therapeutic landscape of CKD. Notably, sodium-glucose co-transporter 2 (SGLT2) inhibitors, originally developed for glycemic control, have emerged as a cornerstone treatment for CKD owing to their ability to reduce intraglomerular pressure and mitigate glomerular hyperfiltration [3, 4]. Additionally, mineralocorticoid receptor antagonists (MRAs) have further diversified the therapeutic options in CKD management. Finerenone, a non-steroidal mineralocorticoid receptor antagonist (ns-MRA), is particularly useful in its ability to slow kidney disease progression and cardiovascular complications in patients with CKD and type 2 diabetes (T2DM) [5]. Major clinical guidelines that endorse the use of SGLT2 inhibitors and MRAs individually, also recognize the potential therapeutic benefits of combining the two regimens. For instance, The Kidney Disease: Improving Global Outcomes (KDIGO) diabetes and CKD clinical practice guidelines recommend adding an ns-MRA to ongoing renin-angiotensin system inhibition and SGLT2 inhibitor therapy to reduce cardiorenal risk in patients with CKD and T2DM who have residual albuminuria [6].

While earlier studies have suggested that combining MRAs with SGLT2 inhibitors offers superior efficacy and safety compared to SGLT2 inhibitor monotherapy, the available evidence remains limited [7]. To address this gap, our updated meta-analysis incorporates high-quality new evidence from the CONFIDENCE trial [8], enabling a more comprehensive and well-powered assessment of the efficacy and safety of combination therapy with MRAs and SGLT2 inhibitors in patients with CKD.

## Methods

This systematic review and meta-analysis was conducted in adherence to the Preferred Reporting Items for Systematic Reviews and Meta-Analyses (PRISMA) guidelines [9], and the review protocol was registered with PROSPERO (CRD420251106173).

### Search strategy

An extensive literature search was independently conducted across PubMed, Cochrane (CENTRAL), ScienceDirect, and Embase targeting randomized controlled trials (RCTs). The search strategy included relevant PubMed entry terms and Medical Subject Headings (MeSH) terms, including (heart failure OR chronic kidney disease OR CKD) AND (mineralocorticoid receptor antagonists OR spironolactone OR finerenone) OR (SGLT2 inhibitors OR empagliflozin OR dapagliflozin) OR (albuminuria OR microalbuminuria OR urinary albumin-to-creatinine ratio). Boolean operators were applied to optimize sensitivity and specificity across all databases. Relevant prior meta-analyses and grey literature were also reviewed to identify research gaps and enhance the inclusion of eligible studies. All databases were searched from their inception until 26 June 2025. To ensure comprehensive coverage, the Embase search was updated on 29 November 2025. The full search strategy for all databases is detailed in Supplementary Table 1, Additional File 1.

### Selection criteria

All identified citations were imported into Rayyan.ai for deduplication [10]. Two reviewers (M.S. and S.L.F.) independently screened titles and abstracts by using predetermined inclusion and exclusion criteria. Full texts of potentially eligible studies were retrieved and assessed for final inclusion. Any disagreements between the two reviewers were first sought to be resolved through discussion and consensus, and if an agreement could not be reached, a third reviewer (A.N.) was consulted to make the final decision. Studies were included if they met the following criteria: (i) published RCTs, (ii) enrolled adult patients with chronic kidney disease (CKD) with or without diabetes, (iii) compared combination therapy of either an MRA or an aldosterone synthase inhibitor (ASI) with an SGLT2 inhibitor versus SGLT2 inhibitor monotherapy, and (iv) reported at least one predefined renal or cardiovascular outcome. Studies were excluded if: (i) they were not RCTs, (ii) they were not published in the English language (iii) lacked relevant outcomes, and (iv) were non-human or non-original studies.

### Data extraction

Two reviewers (S.M.D. and M.R.) extracted data using a structured Excel spreadsheet, including study characteristics, baseline demographics, and outcomes. Primary outcomes included percentage change in albuminuria, change in systolic blood pressure (SBP), change in estimated Glomerular Filtration Rate (eGFR) and change in serum potassium. Secondary outcomes included the proportion of patients with at least a 30% reduction in urinary albumin-to-creatinine ratio (UACR), the proportion of patients with at least a 50% reduction in UACR, hyperkalemia, and any or serious adverse events.

### Risk of bias

Two authors (H.T.G. and F.B.) independently evaluated the included randomised controlled trials using the Revised Cochrane Risk-of-Bias tool for randomized trials (Rob 2.0) [11]. A third author (N.M.K.) reviewed the assessments and resolved any discrepancies.

### Certainty of evidence

The certainty of the evidence for all outcomes was evaluated using the Grading of Recommendations, Assessment, Development, and Evaluations (GRADE) framework [12]. We used the GRADEpro software to generate a summary of findings table, which rated the evidence across the five standard domains: risk of bias, inconsistency, indirectness, imprecision, and publication bias (Supplementary Table 2).

### Data analysis

Meta-analysis was conducted using Review Manager (RevMan) version 5.4.1. Risk ratios (RRs) and mean differences (MDs) were calculated for dichotomous and continuous outcomes, respectively, along with corresponding 95% confidence intervals (CIs). A DerSimonian-Laird random-effects model was used to account for inter-study heterogeneity [13]. Statistical heterogeneity was assessed using the Chi-square test and Higgins’ I² statistic, with I² values interpreted as low (< 25%), moderate (25–75%), or high (> 75%). We conducted subgroup analyses on primary outcomes to determine whether the following factors influenced the effect size: (i) study designs: parallel vs. crossover RCT, (ii) follow-up duration: 4 weeks vs. 12–14 weeks vs. 6 months, and (iii) type of add-on therapy: ns-MRA vs. steroidal mineralocorticoid receptor antagonists (s-MRA) vs. ASI. Sensitivity analysis was also performed using a leave-one-out method to assess the robustness of the findings. A p-value of < 0.05 was considered statistically significant.

## Results

### Study selection

**Fig. 1:**
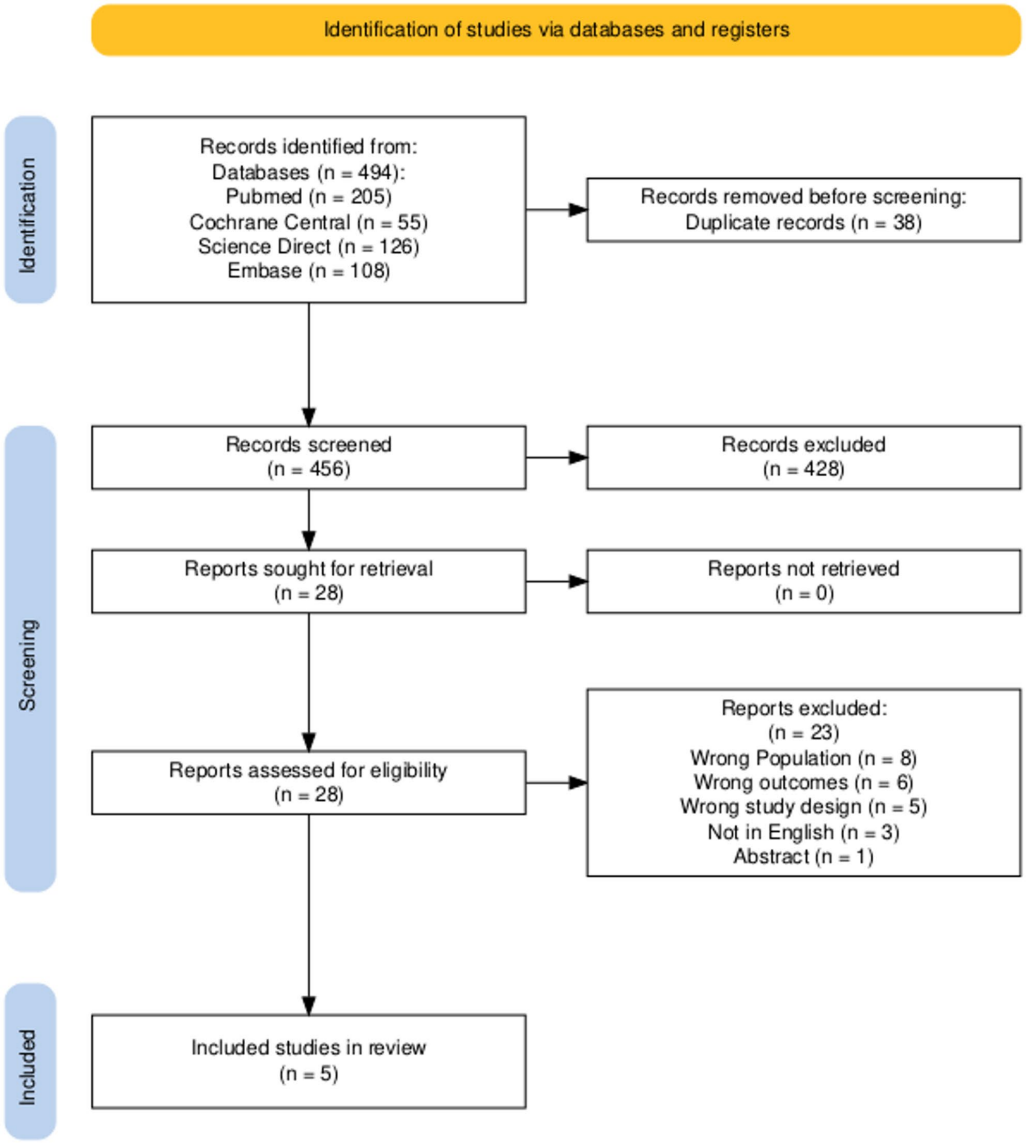
PRISMA flowchart showing the screening and study selection process

After extensive literature search, 494 articles were retrieved from different databases, of which 38 were duplicates. After screening 456 records, 28 studies were identified as potentially eligible. Full-text review of the remaining studies led to the exclusion of 23 additional studies. Finally, 5 randomized controlled trial were considered eligible according to the inclusion criteria and were included in this systematic review and meta-analysis. The PRISMA flow chart summarizes the search and trial selection (Fig. 1).

### Baseline characteristics

A total of 5 RCTs, comprising 808 patients with CKD, were included in this systematic review. These trials compared combination therapy of either an MRA or an ASI with an SGLT2 inhibitor versus SGLT2 inhibitor monotherapy. The ROTATE-3 trial, an open-label crossover study, randomized 46 participants to eplerenone + dapagliflozin or dapagliflozin alone, for a 4-week treatment period, with a mean age of 70 years. The ASi-CKD trial, a double-blind parallel group design, included 148 participants in the vicadrostat 20 mg + empagliflozin 10 mg (*n* = 74) and placebo + empagliflozin 10 mg (*n* = 74), with a mean age of 63 years and a 14-week treatment exposure. The DAPAFINE trial, an open-label design, included 10 participants allocated to dapagliflozin and then to dapagliflozin + finerenone with a mean age of 59 years, and 4-week treatment periods without washout between periods. The MIRACLE trial, a double-blind parallel group design, included 68 participants in balcinrenone 150 mg + dapagliflozin 10 mg (*n* = 35) and placebo + dapagliflozin 10 mg (*n* = 33), with a mean age of 73 years, and 12 weeks of treatment exposure. The CONFIDENCE trial, a double-blind parallel group design, enrolled 536 participants in the finerenone 10 mg or 20 mg + empagliflozin 10 mg (*n* = 269) and empagliflozin 10 mg alone (*n* = 267) with a mean age of 67 years and a 6-month treatment duration. Most patients across the trials were treated with angiotensin converting enzyme inhibitors/angiotensin receptor blockers (ACEis/ARBs) and had type 2 diabetes mellitus, except in the DAPAFINE trial, which excluded patients with diabetes. The baseline characteristics of the included studies are summarized in Table 1.

**Table 1:** Baseline characteristics of patients in the included studies

Characteristics	ROTATE-3	ASi-CKD	DAPAFINE	MIRACLE	CONFIDENCE
Total, N	46	148	10	68	536
Intervention	Eplerenone + Dapagliflozin versus Dapagliflozin	Vicadrostat + Empagliflozin versus placebo + Empagliflozin	Dapagliflozin + Finerenone versus Dapagliflozin	Balcinrenone + Dapagliflozin versus Placebo + Dapagliflozin	Finerenone + Empagliflozin versus Empagliflozin
Type of MRA	sMRA	N/A	ns-MRA	ns-MRA	ns-MRA
Design	Crossover	Parallel group^1^	Crossover	Parallel group^1^	Parallel group
Treatment duration	4 weeks^2^	14 weeks	4 weeks^2^	12 weeks	6 months
Washout	4 weeks	NA	No	NA	NA
Age, years	70 ± 8	63 ± 11	59 ± 15	73 ± 9	67 ± 10
Women, n (%)	11 (24)	51 (35)	3 (30)	18 (26)	137 (25.6)
White race, n (%)	45 (98)	87 (59)	10 (100)	55 (81)	246 (45.9)
T2DM, n (%)	32 (70)	105 (71)	0	44 (65)	536 (100)
SBP, mm Hg	136 ± 9	132 ± 15	130 ± 12	133 ± 18	135 ± 13
BMI, kg/m²	31 ± 6	30 ± 6	25 ± 5	NA	29 ± 6
eGFR, mL/min	58 ± 19	51 ± 17	32 ± 3	43 ± 14	54 ± 16.7
UACR, mg/g	401 (225–629)	391 (192–906)	491 ± 479	87 (13–1234)	578 (287–1056)
ACEi/ARB, n (%)	46 (100)	146 (98)	10 (100)	52 (76)	527 (98.3)
UACR endpoint measure	24-h urine	Spot urine (first morning void)	Spot urine (first morning void) in 2 consecutive days	Spot urine (first morning void)	Spot urine (first morning void) in 2 consecutive days

T2DM, type 2 diabetes mellitus; SBP, systolic blood pressure; BMI, body mass index; eGFR, estimated glomerular filtration rate; UACR, urinary albumin-to-creatinine ratio; ACEi/ARB, angiotensin converting enzyme inhibitor/angiotensin receptor blocker; s-MRA, steroidal mineralocorticoid receptor antagonist; ns-MRA, non-steroidal mineralocorticoid receptor antagonist; NA, not available. ^1^In ASi-CKD, 74 patients were allocated to Vicadrostat 20 mg + empagliflozin 10 mg and 74 patients were allocated to placebo + empagliflozin 10 mg. In MIRACLE, 35 patients were allocated to balcinrenone 150 mg + dapagliflozin 10 mg and 33 patients were allocated to placebo + dapagliflozin 10 mg. In CONFIDENCE, 269 patients were allocated to finerenone 10 mg or 20 mg + empagliflozin 10 mg and 267 patients were allocated to empagliflozin 10 mg. The presented patient characteristics for each of these trials are the average numbers of the randomized groups. ^2^4 weeks for each treatment period

### Risk of bias

**Fig. 2:**
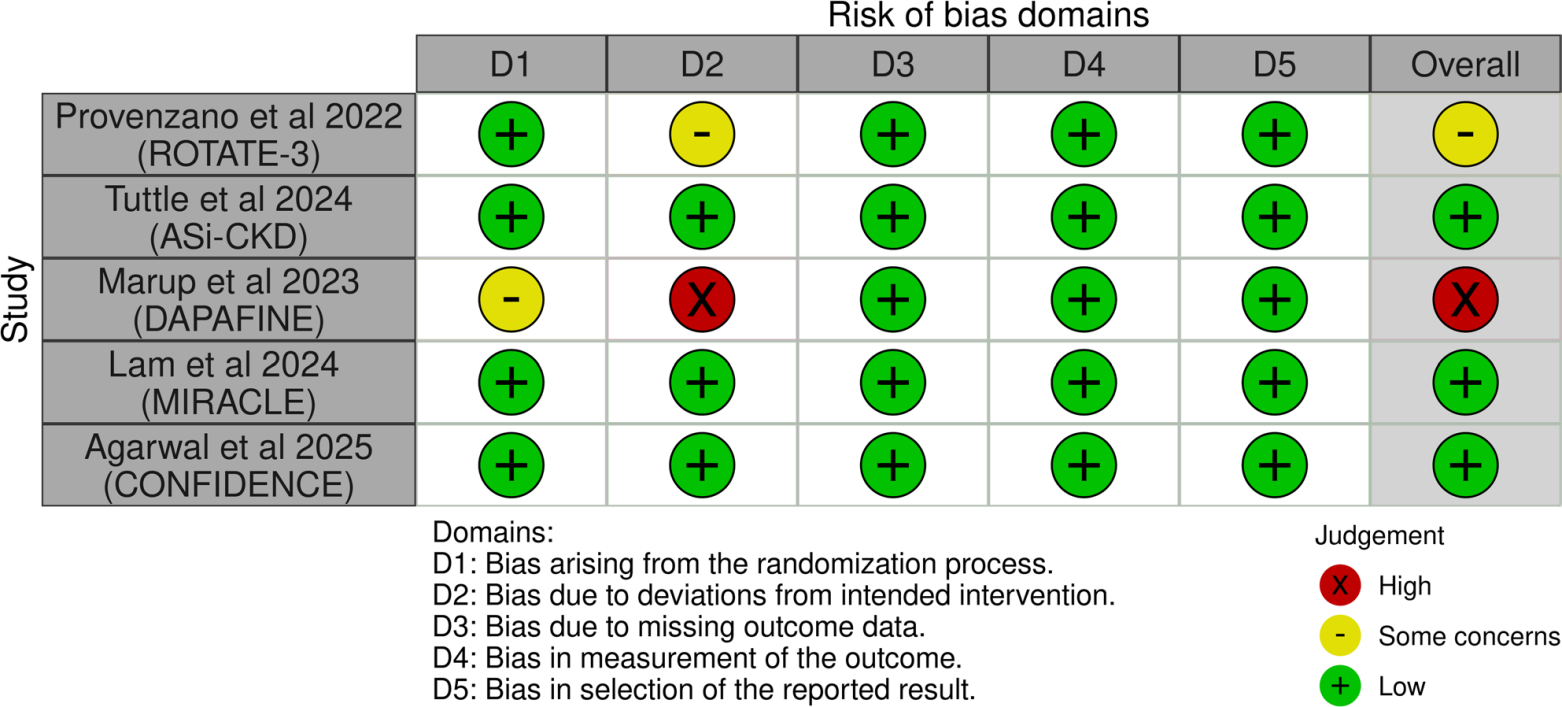
Risk of bias summary for the included trials

**Fig. 3:**
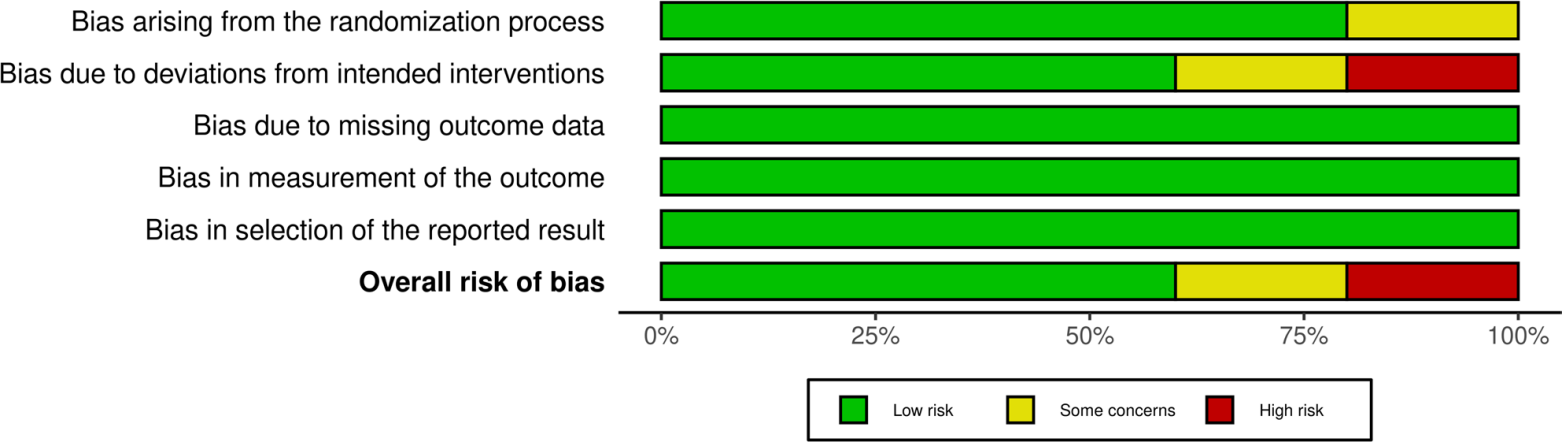
Risk of bias graph for the included trials

Among the five studies, all three parallel group, double-blinded trials (ASi-CKD, MIRACLE, and CONFIDENCE) were assessed as having low risk of bias. Of the two open-label crossover trials, ROTATE-3 was identified as having some concerns due to deviations from the intended interventions, while DAPAFINE was assessed as having high risk of bias due to the absence of a washout period (Figs. 2 and 3).

### Primary outcomes

#### Percentage change in albuminuria (urinary albumin-to-creatinine ratio; UACR)

**Fig. 4:**
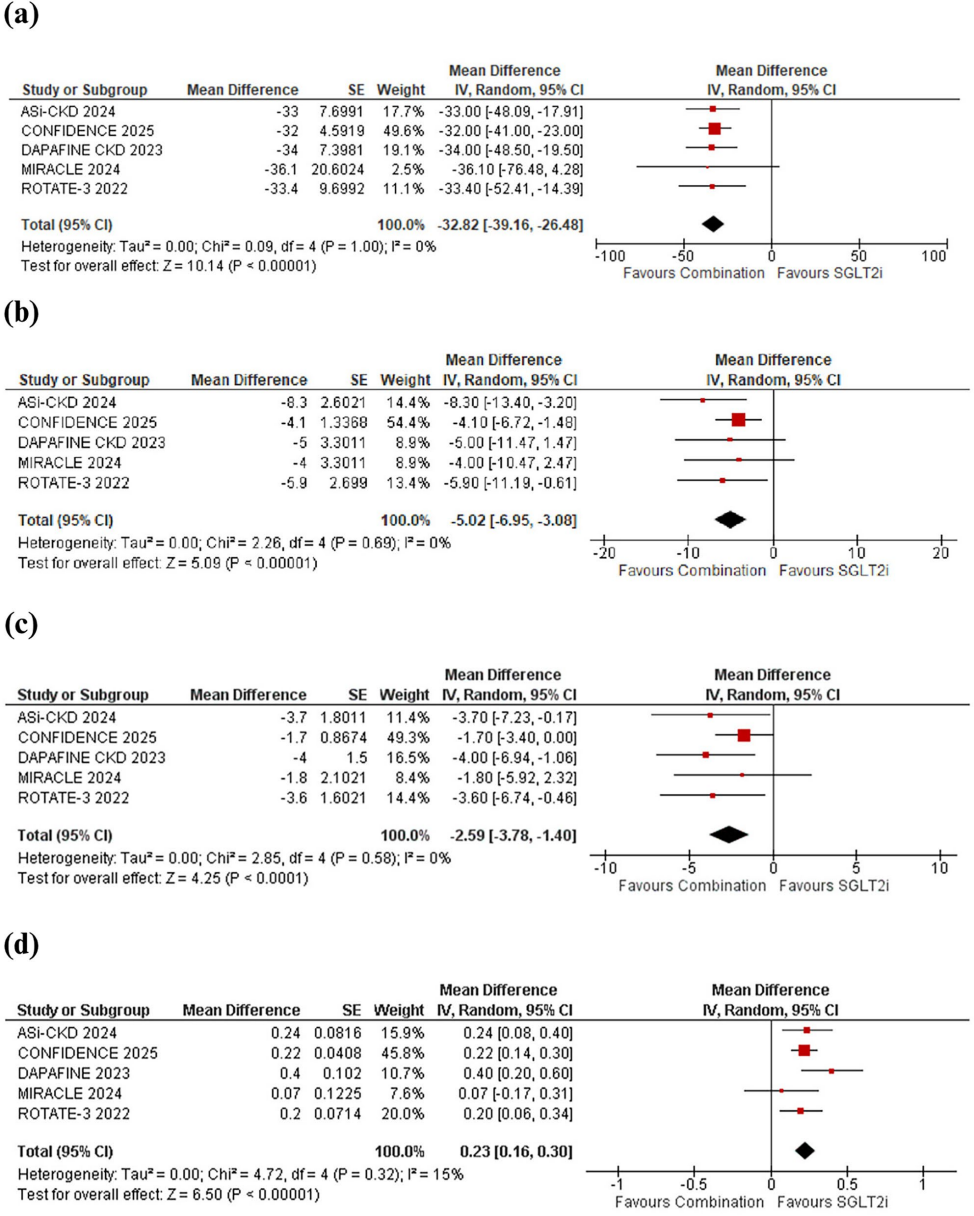
Effect of mineralocorticoid receptor antagonist (MRA) or aldosterone synthase inhibitor (ASI) plus sodium–glucose cotransporter-2 (SGLT2) inhibitor combination therapy versus SGLT2 inhibitor monotherapy on the primary outcomes: (**a**) Percentage change in albuminuria (urinary albumin-to-creatinine ratio; UACR) (**b**) Change in systolic blood pressure (SBP) (**c**) Change in estimated glomerular filtration rate (eGFR) (**d**) Change in serum potassium

Percentage change in albuminuria was reported by 5 studies. Meta-analysis comparing combination therapy with an MRA or ASI plus an SGLT2 inhibitor with SGLT2 inhibitor monotherapy resulted in significant reductions in albuminuria (MD: -32.82% [95% CI: -39.16, -26.48]; *p* < 0.001; I^2^ = 0%; Fig. 4a). Subgroup analysis did not demonstrate effect modification by study design (*p* = 0.84; Supplementary Fig. 1, Additional File 1), follow-up duration (*p* = 0.97; Supplementary Fig. 2, Additional File 1), or type of add-on therapy to SGLT2 inhibitor (*p* = 1.00; Supplementary Fig. 3, Additional File 1).

#### Change in systolic blood pressure (SBP)

Systolic blood pressure (SBP) reduction was reported by 5 studies. Meta-analysis comparing combination therapy with an MRA or ASI plus an SGLT2 inhibitor with SGLT2 inhibitor monotherapy showed a significant reduction in SBP (MD: -5.02 mm Hg [95% CI: -6.95, -3.08]; *p* < 0.001; I² = 0%; Fig. 4b). Subgroup analysis did not demonstrate effect modification by study design (*p* = 0.80; Supplementary Fig. 4, Additional File 1), follow-up duration (*p* = 0.57; Supplementary Fig. 5, Additional File 1), or type of add-on therapy to SGLT2 inhibitor (*p* = 0.33; Supplementary Fig. 6, Additional File 1).

#### Change in estimated glomerular filtration rate (eGFR)

Change in estimated glomerular filtration rate (eGFR) was reported by 5 studies. Meta-analysis showed that combination therapy with an MRA or ASI plus an SGLT2 inhibitor was associated with a greater reduction in eGFR compared with SGLT2 inhibitor monotherapy (MD: -2.59 mL/min/1.73 m^2^ [95% CI: −3.78, − 1.40]; *p* < 0.001; I² = 0%; Fig. 4c). Subgroup analysis did not demonstrate effect modification by study design (*p* = 0.18; Supplementary Fig. 7, Additional File 1), follow-up duration (*p* = 0.31; Supplementary Fig. 8, Additional File 1), or type of add-on therapy to SGLT2 inhibitor (*p* = 0.59; Supplementary Fig. 9, Additional File 1).

#### Change in serum potassium

Meta-analysis showed that combination therapy with an MRA or ASI plus an SGLT2 inhibitor was associated with a greater increase in serum potassium levels compared with SGLT2 inhibitor monotherapy (MD: 0.23 mmol/L [95% CI: 0.16, 0.30]; *p* < 0.001; I² = 15%; Fig. 4d). Subgroup analysis did not demonstrate effect modification by study design (*p* = 0.47; Supplementary Fig. 10, Additional File 1), follow-up duration (*p* = 0.70; Supplementary Fig. 11, Additional File 1), or type of add-on therapy to SGLT2 inhibitor (*p* = 0.91; Supplementary Fig. 12, Additional File 1).

### Secondary outcomes

#### Proportion of patients with at least 30% reduction in UACR

**Fig. 5:**
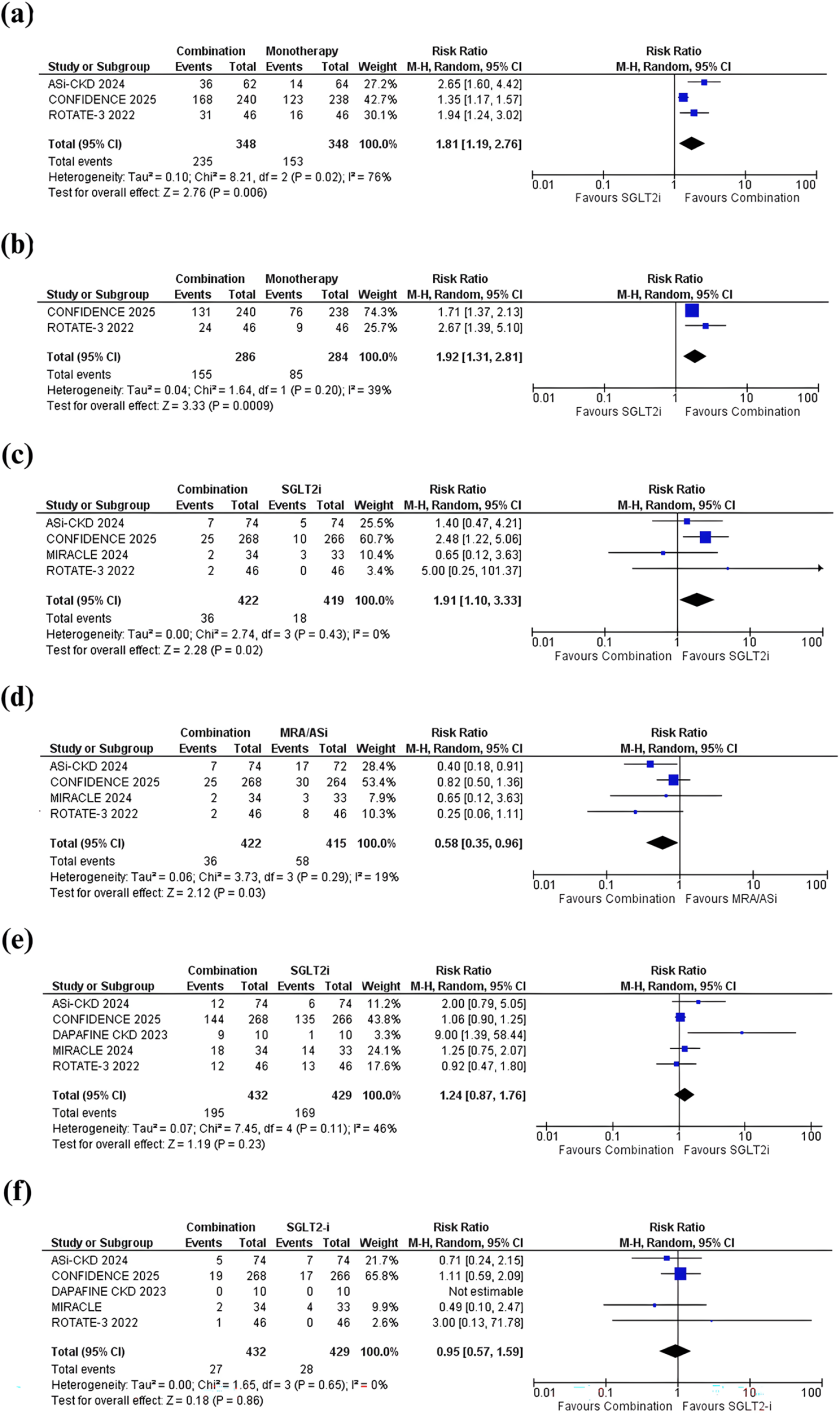
Effect of mineralocorticoid receptor antagonist (MRA) or aldosterone synthase inhibitor (ASI) plus sodium–glucose cotransporter-2 (SGLT2) inhibitor combination therapy versus SGLT2 inhibitor monotherapy on the secondary outcomes: (**a**) Proportion of patients with at least 30% reduction in urinary albumin-to-creatinine ratio (UACR) (**b**) Proportion of patients with at least 50% reduction in UACR (**c**) Hyperkalemia (combination therapy vs. SGLT2 inhibitor monotherapy) (**d**) Hyperkalemia (combination therapy vs. MRA or ASI monotherapy) (**e**) Any adverse events (**f**) Serious adverse events

Meta-analysis of 3 studies showed that, compared with SGLT2 inhibitor monotherapy, combination therapy with an MRA or ASI plus an SGLT2 inhibitor was associated with a significantly greater proportion of patients achieving a ≥ 30% reduction in UACR (RR: 1.81 [95% CI: 1.19, 2.76]; *p* = 0.006; I² = 76%; Fig. 5a). Sensitivity analysis, excluding the CONFIDENCE trial, reduced heterogeneity (I² = 0%) but did not alter the effect size significantly (RR: 2.22; [95% CI: 1.59, 3.10]; *p* < 0.001; Supplementary Fig. 13, Additional File 1).

#### Proportion of patients with at least 50% reduction in UACR

Meta-analysis of 2 studies showed that, in comparison to SGLT2 inhibitor monotherapy, combination therapy with an MRA plus an SGLT2 inhibitor significantly increased the proportion of patients achieving a ≥ 50% reduction in UACR (RR: 1.92 [95% CI: 1.31, 2.81]; *p* < 0.001; I² = 39%; Fig. 5b).

#### Hyperkalemia (MRA or ASI plus SGLT2 inhibitor versus SGLT2 inhibitor alone)

Meta-analysis of 4 studies showed that, in comparison to SGLT2 inhibitor monotherapy, combination therapy with an MRA or ASI plus an SGLT2 inhibitor was associated with a significantly greater risk of hyperkalemia (RR: 1.91 [95% CI: 1.10, 3.33]; *p* = 0.02; I² = 0%; Fig. 5c).

#### Hyperkalemia (MRA or ASI plus SGLT2 inhibitor versus MRA or ASI alone)

Meta-analysis of 4 studies showed that, in comparison to MRA or ASI monotherapy, combination therapy with an MRA or ASI plus an SGLT2 inhibitor was associated with significantly lower rates of hyperkalemia (RR: 0.58 [95% CI: 0.35, 0.96]; *p* = 0.03; I² = 19%; Fig. 5d).

#### Any adverse events

Pooled analysis of 5 studies showed no significant difference in the risk of adverse events between combination therapy with an MRA or ASI plus an SGLT2 inhibitor and SGLT2 inhibitor monotherapy (RR: 1.24 [95% CI: 0.87, 1.76]; *p* = 0.23; I² = 46%; Fig. 5e).

#### Serious adverse events

Pooled analysis of 5 studies showed no significant difference in the risk of serious adverse events between combination therapy with an MRA or ASI plus an SGLT2 inhibitor and SGLT2 inhibitor monotherapy (RR: 0.95 [95% CI: 0.57, 1.59]: *p* = 0.86; I² = 0%; Fig. 5f).

## Discussion

In this meta-analysis encompassing 808 patients with CKD, our analysis revealed multiple clinically significant outcomes. Compared to SGLT2 inhibitor monotherapy, combination therapy with an MRA or ASI plus an SGLT2 inhibitor was associated with significant reductions in both albuminuria and SBP, suggesting synergistic therapeutic effects. This therapeutic combination also yielded a greater reduction in eGFR, in accordance with the well-documented transient phenomenon of eGFR “dip” that often precedes long-term renoprotection [14–16]. Additionally, we found combination therapy to be associated with a modest yet consistent increase in serum potassium levels, attributable to the anti-aldosterone effects of MRA. In terms of safety profile, while the risk of hyperkalemia was elevated with combination therapy compared to SGLT2 inhibitor monotherapy, it was notably lower than that reported with MRA or ASI monotherapy. In comparison to SGLT2 inhibitor monotherapy, combination therapy with an MRA or ASI plus an SGLT2 inhibitor did not differ with respect to total and serious adverse events. Notably, the proportion of patients achieving at least 30% reduction in UACR was the only outcome to demonstrate substantial heterogeneity, primarily influenced by the CONFIDENCE trial. The longer follow-up, higher baseline UACR, and exclusively T2DM population in this study may have contributed to this variability. These factors can affect the magnitude and stability of the treatment effect on UACR, with longer follow-up potentially allowing the effect to decline over time and higher-risk populations tending to show less pronounced reductions.

Our findings, focused on surrogate markers of cardiorenal risk, are consistent with prior literature suggesting additive benefits of combination therapy in CKD [7]. This stands in contrast to a prespecified integrated analysis of 13,000 patients from FIDELIO-DKD (Finerenone in Reducing Kidney Failure and Disease Progression in Diabetic Kidney Disease) and FIGARO-DKD (Finerenone in Reducing Cardiovascular Mortality and Morbidity in Diabetic Kidney Disease) trials, which reported no significant interaction between baseline SGLT2 inhibitor use and finerenone’s effect on hard cardiovascular (CV) and renal endpoints [17]. This discrepancy may be explained by confounding inherent in the non-randomized SGLT2 inhibitor use, as well as the fundamental distinction between a synergistic mechanistic effect on surrogate markers and its clinical translation to hard outcomes. A previous meta-analysis by Ferreira et al. also explored the efficacy of combination therapy with an MRA or ASI plus an SGLT2 inhibitor in patients with CKD but was limited by the inclusion of studies with shorter follow-up durations, smaller sample sizes, and limited range of outcomes, potentially restricting the scope of their findings [7]. Our meta-analysis addressed these limitations by including high quality randomized clinical trials, with larger sample size, longer follow-ups, and greater number of reported outcomes to more comprehensively assess the effectiveness of combination therapy with an MRA or ASI plus an SGLT2 inhibitor in comparison to SGLT2 inhibitor monotherapy in patients with CKD.

The potential mechanistic explanations behind the additive benefits of the combination therapy arise from both distinct and overlapping mechanisms of action between the two regimens. Several large-scale randomized studies have cemented the beneficial effects of SGLT2 inhibitors across a broad spectrum of CKD risk and phenotypes [18–20]. This benefit is attributable, in part, to the multifaceted role of SGLT2 inhibitors in reducing both albuminuria, a key marker for kidney damage, and SBP. In the context of type 2 diabetes mellitus (T2DM), proximal tubular reabsorption of sodium and glucose is markedly increased, which results in a reduced sodium delivery to the macula densa, and the resultant impaired tubuloglomerular feedback [21]. This impaired feedback raises intraglomerular pressure, promoting hyperfiltration and subsequent nephron injury [22–24]. By attenuating glomerular hyperfiltration through restoration of tubuloglomerular feedback, SGLT2 inhibitors interrupt a key pathogenic pathway in CKD progression. These hemodynamic effects, complemented by anti-inflammatory, antifibrotic, and metabolic benefits, underpin their broad renoprotective profile [25].

Similarly, MRA and ASI therapies have proven their efficacy in the context of CKD in patients with and without diabetes. In line with our findings, several preclinical and clinical studies have consistently replicated the beneficial effect of MRAs in reducing albuminuria and systolic blood pressure [17, 26–28]. For instance, in a rat model that developed CKD and endothelial dysfunction due to reduced availability of nitric oxide, finerenone treatment led to a reduction in albuminuria (> 40%) and SBP [29]. The potential mechanistic link behind these observations is provided by the overactivation of mineralocorticoid receptors in non-epithelial cells such as inflammatory cells, podocytes, fibroblasts, mesangial cells, and vascular endothelial cells, which leads to renal inflammation and tubulointerstitial fibrosis [30]. As a consequence of this link, MRAs have surged as an attractive therapeutic option for preventing renal structural injury, reducing albuminuria and preserving renal function [30].

While combination therapy was associated with a higher risk of hyperkalemia compared to SGLT2 inhibitor monotherapy, this risk was still notably attenuated relative to MRA or ASI monotherapy. This risk reduction supports the ‘pillars of care’ model, which posits that combining evidence-based therapies may enhance efficacy while mitigating adverse effects [31]. In the context of a disease as complex as CKD, the varying mechanisms of each of the evidence-based kidney therapies in CKD in patients with and without diabetes implies that a ‘pillars of care’ framework may be the optimal way to address multiple mechanisms of disease progression [32]. This finding regarding hyperkalemia risk reduction with SGLT2 inhibitors has also been reported by other studies previously [33].

All patients included in our meta-analysis had CKD, with over 85% having T2DM. Across the included studies, estimated glomerular filtration rate (eGFR) varied between 30 and 60 mL/min/1.73 m², and urinary albumin-to-creatinine ratio (UACR) ranged from 90 to 600 mg/g. In CKD populations, elevated urinary albumin excretion is a well-established predictor of accelerated eGFR decline [34]. Moreover, given that hypertension affects 80–85% of CKD patients and significantly exacerbates albuminuria progression and cardiovascular risk, dual targeting of albuminuria and blood pressure emerges as a critical priority for disease modification [35, 36].

### Future implications

In a rapidly shifting landscape, several trials are currently underway to clarify the benefits of combination therapy in broader populations, including those with heart failure with preserved ejection fraction (HFpEF) and CKD in patients without diabetes [37–40]. Given our findings, these upcoming trials will be critical in validating the broader applicability and long-term safety of such regimens across diverse CKD phenotypes. Moreover, for patients with T2DM in high or very-high-risk CKD categories, an “accelerated, risk-based approach” to initiation of combination therapy has been proposed [41]. As the evidence base grows, timely integration of guideline-directed combination therapies into clinical practice will be critical to optimizing patient outcomes.

### Limitations

Our meta-analysis has several limitations. First, the findings are based primarily on surrogate endpoints, as data on hard clinical outcomes such as all-cause mortality, cardiovascular mortality, or progression to end-stage kidney disease were not reported in the original trials. Consequently, the impact on these definitive patient-centered outcomes remains uncertain. Second, the pooled population predominantly comprised patients with T2DM, thus the applicability of our findings to CKD populations with other underlying etiologies may be limited. Third, heterogeneity in follow-up durations and types of add-on therapy across studies may have introduced bias, which we addressed with subgroup analyses. Fourth, the lack of a washout period in the DAPAFINE study may have resulted in carryover effects, although this remains speculative. Finally, publication bias could not be formally assessed due to the limited number of studies available for each outcome.

## Conclusion

In summary, this meta-analysis offers robust evidence supporting the additive clinical benefits of combination therapy with an MRA or ASI plus an SGLT2 inhibitor in patients with CKD, particularly in reducing albuminuria and systolic blood pressure. Although a modest increase in serum potassium and risk of hyperkalemia was noted, the overall safety profile remained favourable, especially in comparison to MRA or ASI monotherapy. These findings highlight the therapeutic potential of a multifaceted, mechanism-based approach to CKD management and further substantiate the evolving “pillars of care” framework. Importantly, they also underscore the need for large-scale, prospective studies to evaluate the long-term efficacy of combination therapy in improving hard renal and cardiovascular outcomes.

## Supplementary Information

Below is the link to the electronic supplementary material.

Supplementary Material 1

## Data Availability

The data supporting the findings of this study are based on published literature retrieved from databases including PubMed and Cochrane (CENTRAL). All relevant data extracted from these studies are available in the supplementary material or can be made available upon reasonable request.

## Abbreviations

CKD: Chronic kidney disease
SGLT2: Sodium-Glucose Co-transporter 2
MRAs: Mineralocorticoid Receptor Antagonists
ASIs: Aldosterone Synthase Inhibitors
RCTs: Randomized Controlled Trials
MD: Mean Difference
RR: Risk Ratio
Cis: Confidence Intervals
ns-MRA: Non-steroidal Mineralocorticoid Receptor Antagonists
KDIGO: Kidney Disease: Improving Global Outcomes
PRISMA: Preferred Reporting Items for Systematic Reviews and Meta-Analyses
MeSH: Medical Subject Headings
SBP: Systolic Blood Pressure
eGFR: Estimated Glomerular Filtration Rate
UACR: Urinary Albumin-to-Creatinine Ratio
Rob 2.0: Revised Cochrane Risk-of-Bias tool for randomized trials
RevMan: Review Manager
s-MRA: Steroidal Mineralocorticoid Receptor Antagonists
ACEis: Angiotensin Converting Enzymes Inhibitors
ARBs: Angiotensin Blocking Receptors
FIDELIO-DKD: Finerenone in Reducing Kidney Failure and Disease Progression in Diabetic Kidney Disease
FIGARO-DKD: Finerenone in Reducing Cardiovascular Mortality and Morbidity in Diabetic Kidney Disease
CV: Cardiovascular
T2DM: Type 2 Diabetes Mellitus
HFpEF: Heart Failure with Preserved Ejection Fraction
