# Supplementary material for: Combination therapy with mineralocorticoid receptor antagonists and SGLT2 inhibitors versus SGLT2 inhibitor monotherapy in chronic kidney disease: an updated meta-analysis of randomized controlled trials
Source: BMC Nephrol. 2025 Dec 22;27:69. doi: 10.1186/s12882-025-04710-2 (PMC12837365; doi:10.1186/s12882-025-04710-2)

**Additional File 1**

**Title: Combination Therapy with Mineralocorticoid Receptor Antagonists and SGLT2 Inhibitors Versus SGLT2 Inhibitors Monotherapy in Chronic Kidney Disease: An Updated Meta-Analysis of Randomized Controlled Trials**

**Journal Name**
BMC Nephrology

**Authors**

Shaikh Muhammad Daniyal, MBBS^1^ **ORCID: 0009-0001-4225-0708**
Hareem Ajaz, MBBS^1^ **ORCID: 0009-0000-4268-6686**

Minahil Riaz, MBBS^1^ **ORCID: 0009-0009-3454-3189**

Naveen Murad Khatoon, MBBS^1^ **ORCID: 0009-0003-4948-9994**

Zunaira Aftab, MBBS^1^ **ORCID:** **0009-0003-7279-7266**

Habiba Tauqir Gondal, MBBS^1^ **ORCID: 0009-0001-0681-0042**

Isbah Gul, MBBS^1^ **ORCID: 0009-0005-0803-3493**

Mahwish Sarwar, MBBS^1^ **ORCID: 0009-0000-0518-4900**
Fizza Batool, MBBS^1^ **ORCID: 0009-0007-7669-9451**

Syeda Laiba Fahim, MBBS^1^ **ORCID: 0009-0001-5819-0498**

Amna Noor, MBBS^1^ **ORCID: 0009-0009-2791-9213**

Ayan Khalid, MBBS^1^ **ORCID: 0009-0007-1928-7100**

Danish Ali Ashraf, MBBS^2^ **ORCID: 0000-0002-3013-9359**

Romal Jabarkhil, MBBS^3^ **ORCID: 0009-0006-7339-7100**

**Affiliations**

^1^ Department of Medicine, Dow University of Health Sciences, Karachi, Pakistan

^2^ Department of Medicine, TruGift Health LLC, Wilmington, Delaware, USA

^3^ Ningarhar Regional Hospital: Jalalabad, Afghanistan, AF

**Corresponding Author**

Romal Jabarkhil

Department of Medicine

Ningarhar Regional Hospital: Jalalabad, Afghanistan, AF

Email Address: [romaljabarkhil@outlook.com](mailto:romaljabarkhil@outlook.com)

**Supplementary Table 1** Detailed search strategies used along with records retrieved

| **Database or Search Engine** | **String** | **Results** |
| --- | --- | --- |
| Pubmed | ("Heart Failure"[MeSH] OR "Renal Insufficiency, Chronic"[MeSH] OR "chronic kidney insufficiency" OR "chronic kidney disease" OR CKD) AND ("Mineralocorticoid Receptor Antagonists"[MeSH] OR mineralocorticoid receptor antagonists OR Empagliflozin OR Dapagliflozin OR eplerenone OR canrenone OR spironolactone OR finerenone OR aldosterone synthase inhibitor OR Esaxerenone OR Baxdrostat) AND ("Sodium-Glucose Transporter 2 Inhibitors"[MeSH] OR canagliflozin OR dapagliflozin OR empagliflozin OR ertugliflozin) AND ("Albuminuria"[MeSH] OR albuminuria OR microalbuminuria OR "urinary albumin-to-creatinine ratio") | 205 |
| Cochrane | (heart failure OR chronic kidney disease OR chronic kidney insufficiency OR renal insufficiency OR CKD) AND (mineralocorticoid receptor antagonists OR aldosterone antagonists OR eplerenone OR spironolactone OR finerenone OR canrenone OR esaxerenone OR baxdrostat OR aldosterone synthase inhibitor) AND (SGLT2 inhibitors OR empagliflozin OR dapagliflozin OR canagliflozin OR ertugliflozin) AND (albuminuria OR microalbuminuria OR urinary albumin-to-creatinine ratio OR UACR) | 55 |
| ScienceDirect | "chronic kidney disease" AND "mineralocorticoid receptor antagonists" AND "SGLT2 inhibitors" AND "microalbuminuria" | 126 |
| Embase | ('chronic kidney disease'/exp OR 'chronic kidney insufficiency' OR 'chronic renal insufficiency' OR 'chronic kidney disease':ti,ab OR CKD:ti,ab) AND ('mineralocorticoid receptor antagonist'/exp OR 'mineralocorticoid receptor antagonist*':ti,ab OR eplerenone:ti,ab OR spironolactone:ti,ab OR canrenone:ti,ab OR finerenone:ti,ab OR esaxerenone:ti,ab OR baxdrostat:ti,ab  OR 'aldosterone synthase inhibitor*':ti,ab) AND ('sodium glucose transporter 2 inhibitor'/exp OR 'SGLT2 inhibitor*':ti,ab OR canagliflozin:ti,ab OR dapagliflozin:ti,ab OR empagliflozin:ti,ab OR ertugliflozin:ti,ab) AND ('albuminuria'/exp OR albuminuria:ti,ab OR microalbuminuria:ti,ab OR 'urinary albumin-to-creatinine ratio':ti,ab OR 'albumin creatinine ratio':ti,ab OR UACR:ti,ab) | 108 |

**Supplementary Table 2** Grade assessment table

| **Certainty assessment** | | | | | | | **Summary of findings** | | | | |
| --- | --- | --- | --- | --- | --- | --- | --- | --- | --- | --- | --- |
| **Participants (studies) Follow-up** | **Risk of bias** | **Incons**  **Isten**  **cy** | **Indirectness** | **Imprecision** | **Publication bias** | **Overall certainty of evidence** | **Study event rates (%)** | | **Relative effect (95% CI)** | **Anticipated absolute effects** | |
|  |  |  |  |  |  |  | **With SGLT2 Inhibitors Monotherapy** | **With Mineralocorticoid Receptor Antagonists and SGLT2 Inhibitors** |  | **Risk with SGLT2 Inhibitors Monotherapy** | **Risk difference with Mineralocorticoid Receptor Antagonists and SGLT2 Inhibitors** |
| **Percentage change in albuminuria (UACR %)** | | | | | | | | | | | |
| 864 (5 RCTs) | serious^a^ | not serious | not serious | not serious | none | ⨁⨁⨁◯ Moderate^a^ | 430 | 434 | - | 430 | MD **32.82 lower** (39.16 lower to 26.48 lower) |
| **Change in systolic blood pressure (SBP)** | | | | | | | | | | | |
| 864 (5 RCTs) | serious^a^ | not serious | not serious | not serious | none | ⨁⨁⨁◯ Moderate^a^ | 430 | 434 | - | 430 | MD **5.02 lower** (6.95 lower to 3.08 lower) |
| **Change in estimated glomerular filtration rate (eGFR)** | | | | | | | | | | | |
| 864 (5 RCTs) | serious^a^ | not serious | not serious | not serious | none | ⨁⨁⨁◯ Moderate^a^ | 430 | 434 | - | 430 | MD **2.59 lower** (3.78 lower to 1.4 lower) |
| **Hyperkalemia (combination vs SGLT2 inhibitors alone)** | | | | | | | | | | | |
| 841 (4 RCTs) | serious^a^ | not serious | not serious | not serious | none | ⨁⨁⨁◯ Moderate^a^ | 18/419 (4.3%) | 36/422 (8.5%) | **RR 1.91** (1.10 to 3.33) | 18/419 (4.3%) | **39 more per 1,000** (from 4 more to 100 more) |
| **Any adverse events** | | | | | | | | | | | |
| 861 (5 RCTs) | serious^a^ | not serious | not serious | not serious | none | ⨁⨁⨁◯ Moderate^a^ | 169/429 (39.4%) | 195/432 (45.1%) | **RR 1.24** (0.87 to 1.76) | 169/429 (39.4%) | **95 more per 1,000** (from 51 fewer to 299 more) |
| **Serious adverse events** | | | | | | | | | | | |
| 861 (5 RCTs) | serious^a^ | not serious | not serious | not serious | none | ⨁⨁⨁◯ Moderate^a^ | 28/429 (6.5%) | 27/432 (6.3%) | **RR 0.95** (0.57 to 1.59) | 28/429 (6.5%) | **3 fewer per 1,000** (from 28 fewer to 39 more) |
| **Proportion of patients achieving ≥30% reduction in UACR** | | | | | | | | | | | |
| 570 (2 RCTs) | not serious | not serious | not serious | not serious | none | ⨁⨁⨁⨁ High | 85/284 (29.9%) | 155/286 (54.2%) | **RR 1.92** (1.31 to 2.81) | 85/284 (29.9%) | **275 more per 1,000** (from 93 more to 542 more) |

***CI:*** *confidence interval;* ***MD:*** *mean difference;* ***RR:*** *risk ratio. ^a^Few studies have some concern and high ROB*

**Supplementary Fig. 1** Subgroup analysis based on study design for percentage change in albuminuria (urinary albumin-to-creatinine ratio; UACR)


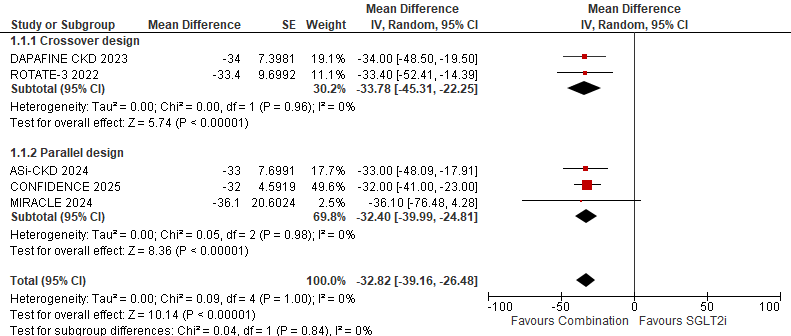


**Supplementary Fig. 2** Subgroup analysis based on follow-up for percentage change in albuminuria (urinary albumin-to-creatinine ratio; UACR)


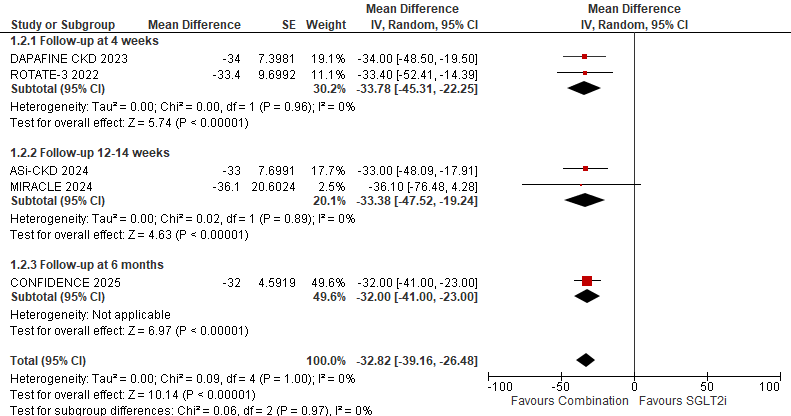


**Supplementary Fig. 3** Subgroup analysis based on type of add-on therapy for percentage change in albuminuria (urinary albumin-to-creatinine ratio; UACR)


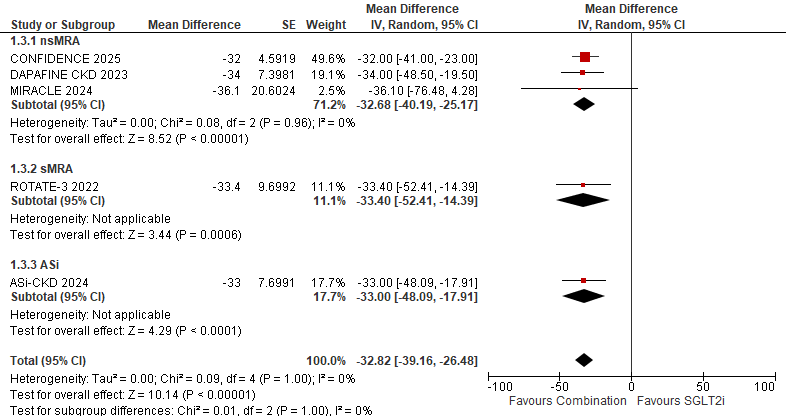


**Supplementary Fig. 4** Subgroup analysis based on study design for change in systolic blood pressure (SBP)


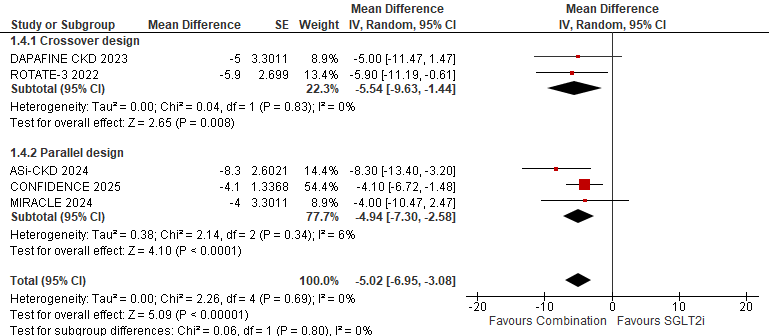


**Supplementary Fig. 5** Subgroup analysis based on follow-up for change in systolic blood pressure (SBP)


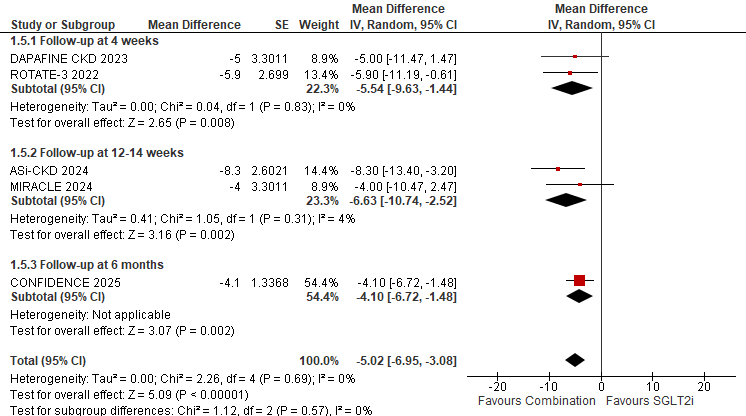


**Supplementary Fig. 6** Subgroup analysis based on type of add-on therapy for change in systolic blood pressure (SBP)


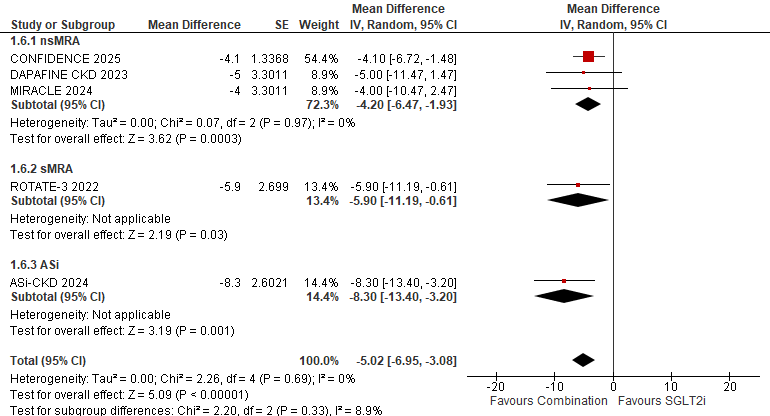


**Supplementary Fig. 7** Subgroup analysis based on study design for change in effective glomerular filtration rate (eGFR)


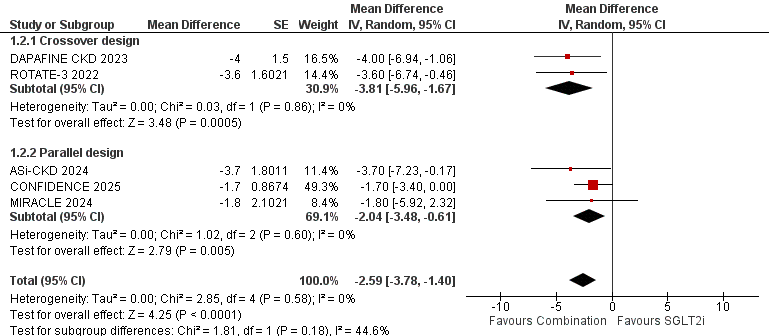


**Supplementary Fig. 8** Subgroup analysis based on follow-up for change in effective glomerular filtration rate (eGFR)


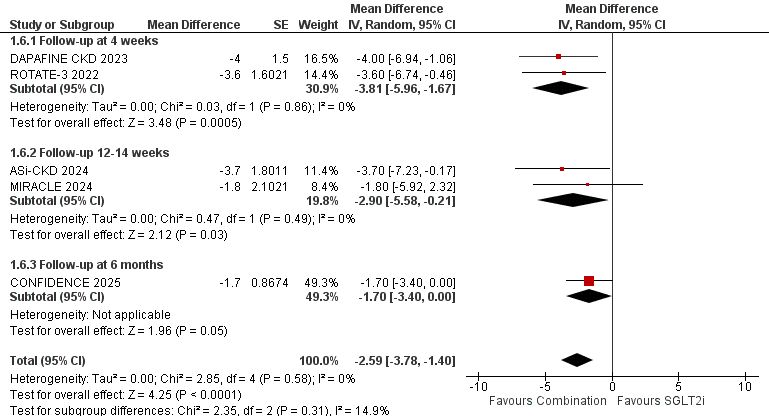


**Supplementary Fig. 9** Subgroup analysis based on type of add-on therapy for change in effective glomerular filtration rate (eGFR)


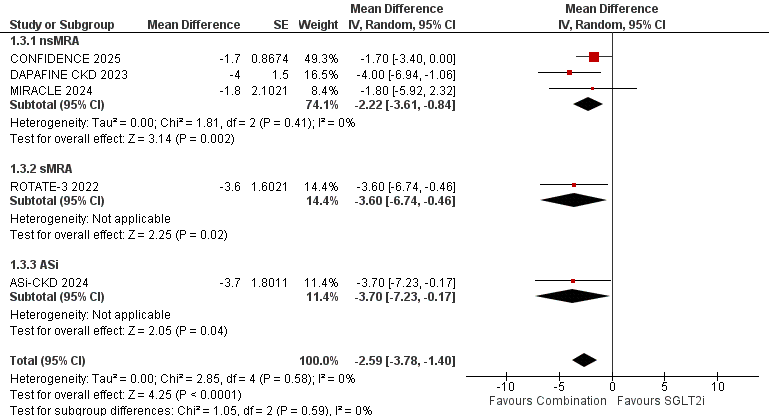


**Supplementary Fig. 10** Subgroup analysis based on study-design for change in serum potassium levels


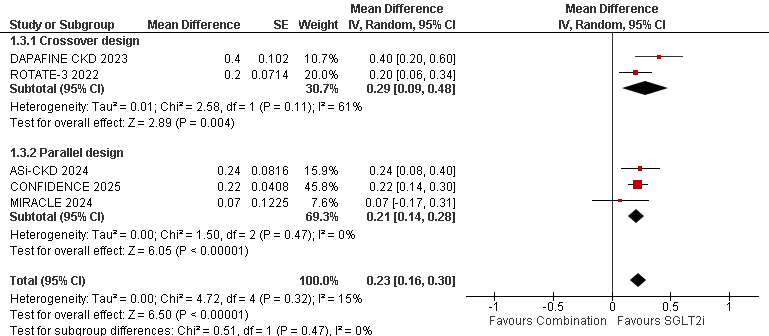


**Supplementary Fig. 11** Subgroup analysis based on follow-up for change in serum potassium levels


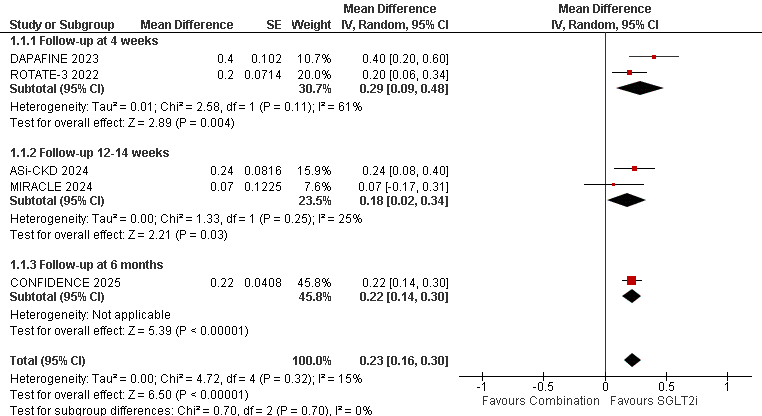


**Supplementary Fig. 12** Subgroup analysis based on type of add-on therapy for change in serum potassium levels


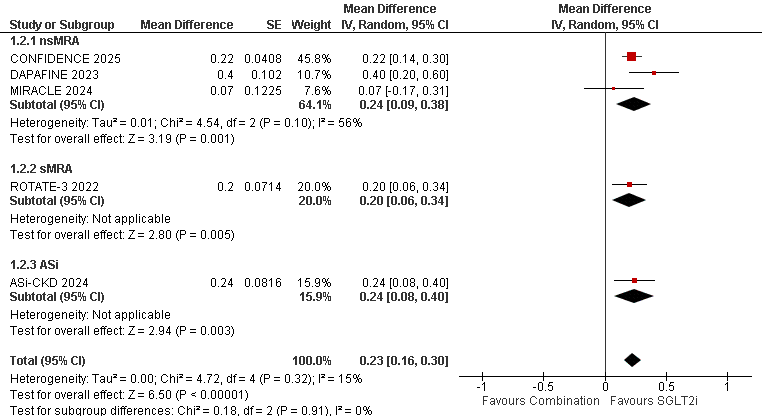


**Supplementary Fig. 13** sensitivity analysis excluding “CONFIDENCE 2025” study for proportion of patients with at least 30% reduction in UACR


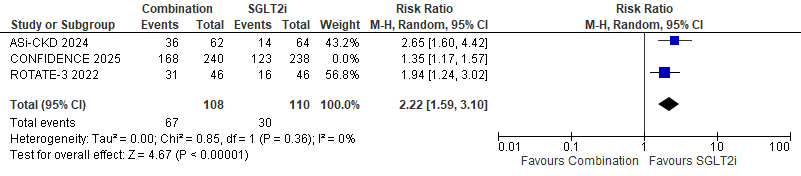

Supplement: Supplementary file 1 — Supplementary Material 1 [file 12882_2025_4710_MOESM1_ESM.docx]
